# Glycan Dependence of Galectin-3 Self-Association Properties

**DOI:** 10.1371/journal.pone.0111836

**Published:** 2014-11-04

**Authors:** Hubert Halimi, Annafrancesca Rigato, Deborah Byrne, Géraldine Ferracci, Corinne Sebban-Kreuzer, Latifa ElAntak, Francoise Guerlesquin

**Affiliations:** 1 Laboratoire d'Ingénierie des Systèmes Moléculaires, UMR 7255, CNRS, Aix-Marseille Université, Marseille, France; 2 U1006 INSERM, Aix-Marseille Université, Marseille, France; 3 Institut de la Microbiologie de la Méditerranée, FR 3479, CNRS, Aix-Marseille Université, Marseille, France; 4 Centre de Recherche en Neurobiologie et Neurophysiologie de Marseille, UMR 7286, Aix-Marseille Université, Marseille, France; University of Liverpool, United Kingdom

## Abstract

Human Galectin-3 is found in the nucleus, the cytoplasm and at the cell surface. This lectin is constituted of two domains: an unfolded N-terminal domain and a C-terminal Carbohydrate Recognition Domain (CRD). There are still uncertainties about the relationship between the quaternary structure of Galectin-3 and its carbohydrate binding properties. Two types of self-association have been described for this lectin: a C-type self-association and a N-type self-association. Herein, we have analyzed Galectin-3 oligomerization by Dynamic Light Scattering using both the recombinant CRD and the full length lectin. Our results proved that LNnT induces N-type self-association of full length Galectin-3. Moreover, from Nuclear Magnetic Resonance (NMR) and Surface Plasmon Resonance experiments, we observed no significant specificity or affinity variations for carbohydrates related to the presence of the N-terminal domain of Galectin-3. NMR mapping clearly established that the N-terminal domain interacts with the CRD. We propose that LNnT induces a release of the N-terminal domain resulting in the glycan-dependent self-association of Galectin-3 through N-terminal domain interactions.

## Introduction

Galectins are small soluble lectins having carbohydrate-dependent extracellular and carbohydrate-independent intracellular activities [1]. Oligomerization is one of the unique features of secreted galectins forming ordered galectin-glycan lattices at the cell surface. Fourteen members of the galectin family have been identified in mammals and designated as galectin-1 to galectin-14. The common property of galectins is the presence of a carbohydrate-recognition domain (CRD) of about 130 amino acids with a highly conserved folding that confers affinity for β-galactoside-containing glycans [1]. Galectins are classified in three categories according to their structures [1]. The members of the prototype family which exist as monomers or homodimers and contain one CRD per subunit are Galectin-1, -2, -5, -7, -10, -11, -13 and -14. The tandem-repeat family accommodates two CRD domains in a single polypeptide chain connected by a non-conserved linker sequence of up to 70 amino acids. This family is composed of Galectin-4, -6, -8, -9 and -12. Galectin-3 is the unique member of the chimera family and its single polypeptide chain forms two distinct domains, a non-lectin N-terminal domain and a C-terminal domain constituting the CRD. Galectin-3 N-terminal domain encloses a short N-terminal segment necessary for secretion followed by collagen-like repeats connected to the C-terminal CRD domain [2].

Galectin-3 displays a large range of cellular locations [3]. It is found in the nucleus, the cytoplasm and can be secreted *via* a non-classical pathway outside the cell [4]. In adults, Galectin-3 is ubiquitously distributed in tissues and it is thus involved in a large number of physiological and pathological processes such as cell proliferation, cell differentiation, cell survival and cell death [5]–[6]. Galectin-3 N-terminal domain is essential for its biological activities [2]. This unstructured domain is subject to metalloproteinase proteolysis, which impacts on its biological functions. Cleavage at different positions has been shown to be a fine tuning of Galectin-3 activity and specificity. Serine and tyrosine phosphorylations have been described as post-translational modifications [7]. These modifications are involved in Galectin-3 localization by regulating collagen domain cleavage [8]. Galectin-3 has also an extracellular localization at the cell surfaces or in the extracellular matrix [4]. This lectin has been shown to mediate cell adhesion involving cell surface glycosylated components. Extracellular lattice formation resulting of Galectin-3/glycan interactions is an interesting problem, but a lot of contradictory structural information is found in the literature. The role of Galectin-3 N-terminal domain was suggested to be either in modulating the affinity of the lectin for carbohydrates or regulating the self-association of this chimera lectin. The CRD is responsible for the lectin activity of Galectin-3 [9], but the implication of the N-terminal domain in carbohydrate interaction is still an open question. Moreover, two models of Galectin-3 self-association have been reported: a C-type self-association involving the CRD [10] and a N-type self-association involving the N-terminal domain [11]. It has been reported that Galectin-3 precipitates as a pentamer with synthetic multivalent carbohydrates and forms disorganized heterogeneous cross-linked complexes [12].

Using recombinant full length Galectin-3 (FL) and Galectin-3 CRD (CRD), we have investigated by Dynamic Light Scattering (DLS) the self-association properties of Galectin-3 in the presence or the absence of carbohydrates. A mechanism for the ligand-induced N-type association is proposed on the basis of our structural NMR data on CRD/N-terminal domain interactions in the presence or the absence of the lacto-N-neoTetraose (LNnT).

## Material and Methods

### Protein expression

The cDNA sequences encoding N-terminal domain (13–113), CRD domain (114–250) and full length (1–250) human Galectin-3 were PCR amplified using appropriate primers (Table S1) in order to include a 6His-tag sequence at the N-terminus for the N-terminal domain and at the C-terminus for CRD and full length protein. The encoding sequences were then cloned into a pET22b(+) expression vector. After transformation of BL21(DE3) competent cells, the bacteria were grown on minimum cell medium M9 at 37°C until OD_600 nm_ = 0.6. Then IPTG 1 mM was added to the culture to induce overexpression of the proteins. After 4 hours at 37°C, cells were harvested. From a French press cell lysate, the His-Tag proteins were purified from the supernatant by affinity chromatography using a HiTrap pre-packed column on an AKTAPrime purifying system (GE Healthcare). Proteins were eluted using a linear imidazole gradient and dialyzed in a 5 mM potassium phosphate buffer at pH 7.4. EDTA was added to purify fractions of N-terminal domain and full length Galectin-3 to avoid cleavage by metalloproteinases. Protein purity was checked by coomassie blue staining of a SDS-PAGE and mass spectrometry analysis. For isotopic labeling, ^15^N-ammonium chloride was used as the sole source of nitrogen and ^13^C-C_6_glucose was used as the sole source of carbon.

### NMR experiments

NMR experiments were carried out at 303 K on a Bruker Avance III 600 MHz NMR spectrometer equipped with a TCI cryoprobe, and a Bruker Avance III 500 MHz NMR spectrometer. CRD chemical shift assignments were obtained from [13] and two sets of heteronuclear NMR experiments (HNCA and HNCOCA, and HNCO and HNCACO) were performed on the ^15^N and ^13^C-labeled CRD sample at 400 mM concentration in the presence and the absence of lactose. ^1^H-^15^N-HSQC titrations were performed on ^15^N-labeled proteins at 40 µM concentration for CRD and 30 µM for full length protein (FL), in the absence and the presence of various concentrations of ligands (lactose or LNnT). All NMR experiments were performed on samples in 5 mM potassium phosphate buffer at pH 7.4. Lactose was purchased from Sigma and LNnT from Elicityl Company.

### Surface Plasmon Resonance experiments

SPR experiments were performed at 25°C on a NiHC sensor chip (Xantec) with a Biacore T200 instrument (GE Healthcare) using 10 mM HEPES-NaOH pH 7.4, 150 mM NaCl, 50 µM EDTA, 0.005% Tween-20 as the running buffer. Six his-tagged CRD and FL were immobilized (230 fmoles) by affinity on two independent experimental flow cells, and two flow cells without protein were used as control. A set of concentrations of lactose and LNnT were successively injected over all flow cells at a flow rate of 30 µl/min during one minute. Sensorgrams obtained from control flow cells were systematically subtracted from those obtained over CRD and FL. The K_D_ values were calculated by plotting saturation binding curves using the equilibrium response at the plateau of all curves with BiaEvaluation software version 2.0 (GE Healthcare). Each value was obtained from at least two independent experiments performed in triplicate.

### Dynamic Light Scattering

We performed Dynamic Light Scattering (DLS) experiments using a Zetazizer Nano Series (Malvern Instruments, London, UK). The samples were analyzed in a disposable micro-cuvette ZEN0040. The samples were measured in triplicate. Each measurement consisted of 11 runs, each run lasting for 10 seconds. We used a laser He-Ne at 633 nm with a scattering detection angle of 173°. All analyses were performed at 25°C. The solutions containing 41 µM CRD and 37 µM FL, in 5 mM potassium phosphate buffer at pH 7.4, were centrifuged at 14,000 rpm for 5 minutes and filtered through a 0.45 µm filter. Sodium phosphate buffer with a viscosity of 0.89 cp and a refractive index of 1.33 was used for all sample preparations. We used the standard refractive index 1.45 for a spherical protein to calculate the mass distribution of size. Protein analysis was performed using the instrument software based on the model determined from an L.curve. We used a standard operating procedure for protein analysis. All measurement conditions were optimized automatically by the instruments software. We added increments of 1 µl of 100 mM lactose to a 50 µl sample containing CRD and FL respectively. The concentrations of lactose used were from 2 mM to 8 mM. LNnT was added in 0.5 µl increments of a 7 mM stock to 50 µl sample containing CRD and FL respectively. The LNnT concentrations added to the FL sample were from 70 µM to 210 µM, in contrast to the higher amounts added to CRD, 70 µM to 1.2 mM.

## Results and Discussion

### Oligomerization states of Galectin-3 investigated by DLS

**Figure 1:**
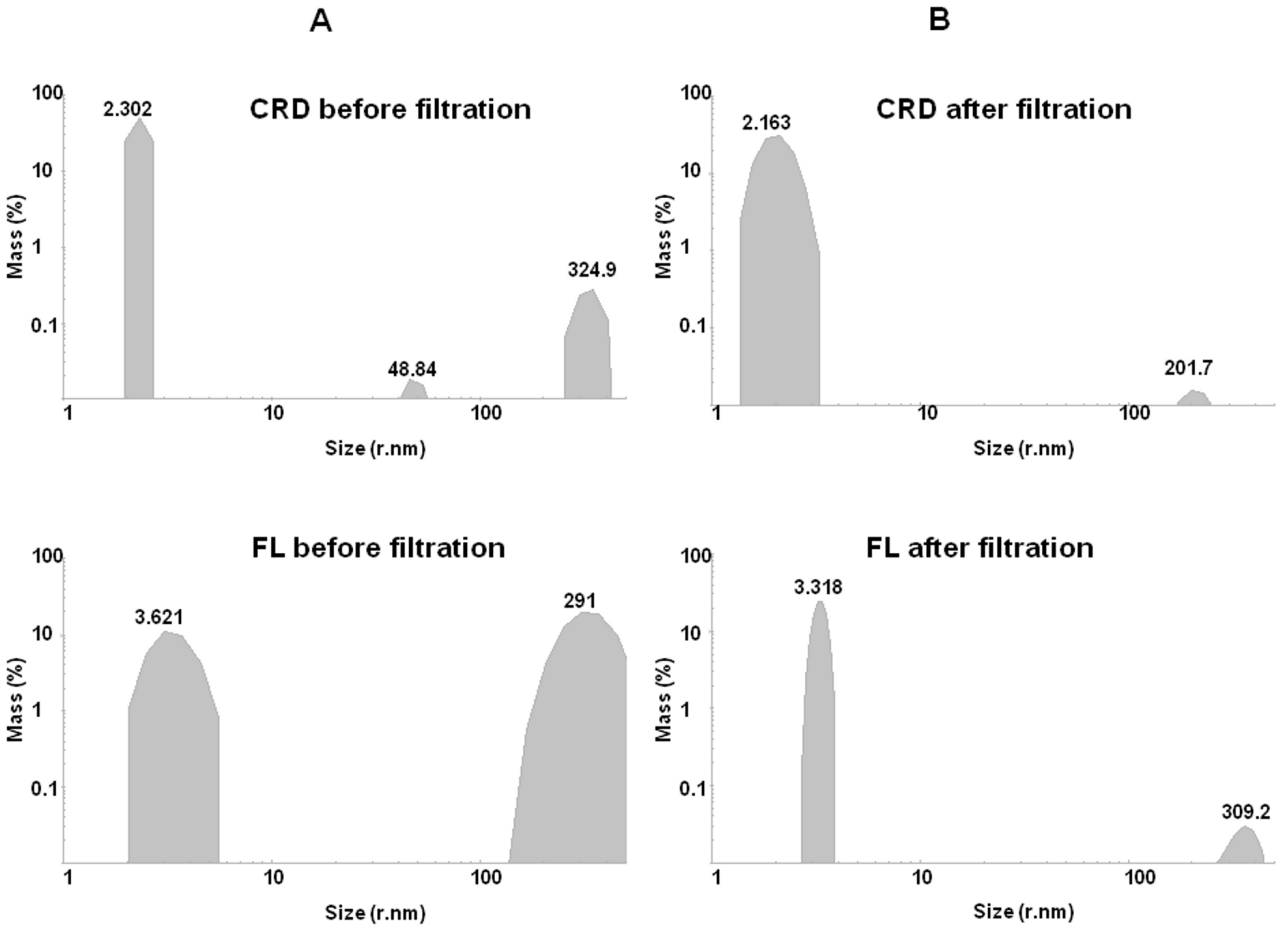
CRD and FL oligomerization states without glycan determined by DLS. Top, CRD (41 µM) size distribution by volume before and after filtration. Bottom, FL (37 µM) size distribution by volume before and after filtration.

**Figure 2:**
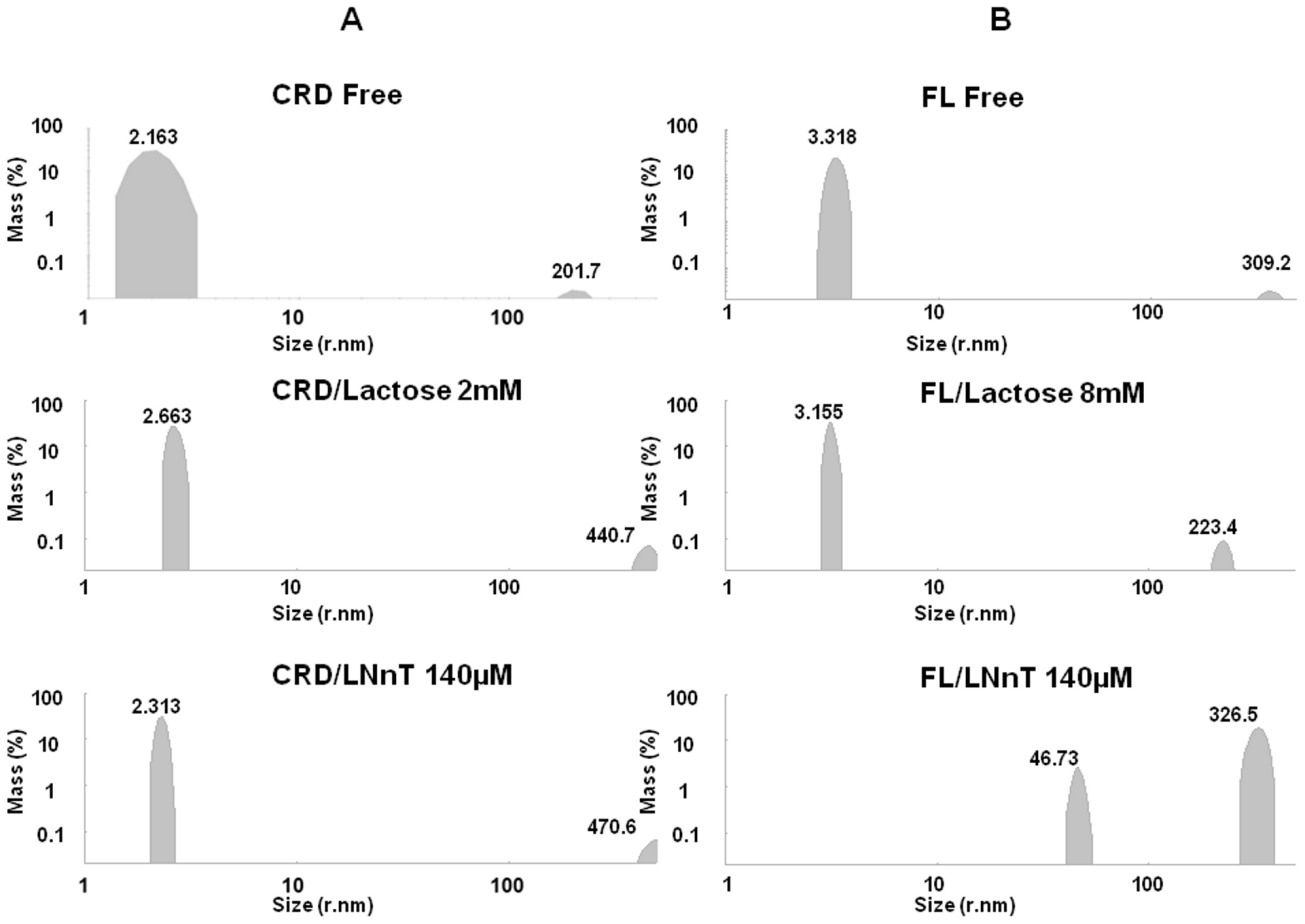
CRD and FL glycan induced oligomerization determined by DLS. A] CRD (41 µM) size distribution by volume before and after the addition of glycans. Top, monomeric CRD without glycan; middle, monomeric CRD with 2 mM lactose; bottom, monomeric CRD with 140 µM LNnT. B] FL (37 µM) size distribution by volume before and after the addition of glycans. Top, FL monomer without glycan; middle, FL monomer with 8 mM lactose; bottom, FL oligomers with 140 µM LNnT.

Taking into account the large number of contradictory results already published in the literature regarding the oligomeric states of Galectin-3, our first concern was to investigate the oligomerization states of CRD and full length Galectin-3 by DLS. After purification and freezing at −80°C, we could observe that both CRD and FL were present in two states: a small hydrodynamic size as already reported in the literature and a large hydrodynamic size, indicating an oligomerization of the Galectin-3 independent of the glycan presence and independent of the N-terminal domain, corresponding to the C-type association (Table 1 and Fig. 1). However, when comparing FL and CRD samples, it appeared that FL was more sensitive to oligomerization. We optimized a protocol including centrifugation (14,000 rpm during 5 mn) and filtering (0.45 µm) which allowed us to obtain a sample predominantly constituted of molecules with a hydrodynamic radius less than 5 nm (Table 1), consistent with the monomeric form of the lectin. We observed that the monomeric state of CRD or FL samples remained stable at room temperature for several hours following filtration. The samples also remained stable up to 47°C. With the aim to observe glycan induced oligomerization, we have performed all the DLS experiments using samples centrifuged, filtered and analyzed at room temperature. Two carbohydrates were tested, lactose, well established as a good ligand for Galectin-3 and LNnT [14]. Both x-ray structures of the CRD/lactose complex (PDB 3ZSJ) and CRD/LNnT complex (PDB 4LBN) have been solved. In Table 1, the results of DLS experiments are summarized, showing the percentages of different hydrodynamic sizes (corresponding to different oligomerization states) for both CRD and FL in the absence and presence of the ligands. The comparison between the initial percentage of monomeric CRD and FL (size <5 nm) and after lactose addition, clearly shows that lactose does not induce CRD nor FL oligomerization (Fig. 2). This data is in agreement with previous data found in the literature [15]. Interestingly, with the addition of 70 µM LNnT to the FL solution, two populations were present; the first with a hydrodynamic radius of 3.147 nm representing 66.7% of the total mass and the second with a radius of 4.643 nm, representing 33.3% (Table 1, sample 9). The 4.643 nm population could be indicative of a pentameric state already described in the literature (Ahmad et al, 2004). Consequently, when we increased the concentration two fold of LNnT, the percentage of protein with low hydrodynamic size (less than 5 nm) dropped to zero, while an increase of two populations with larger hydrodynamic radii was observed. The first population with a hydrodynamic radius greater than 40 nm, represents 8.7% of the total mass and the second population with a radius greater than 100 nm, represents 91.3%. On the contrary, the presence of LNnT induced no effects on the CRD. The evidence clearly demonstrates that LNnT is able to induce oligomerization of the full-length protein, but not of the CRD alone. Such results strongly support the hypothesis of an essential role of the N-terminal domain in the mechanism of ligand-dependent oligomerization of Galectin-3 *via* an N-type self-association as recently reported in the literature [11].

**Table 1:** Percentage distribution of size in hydrodynamic radius of CRD and FL in the presence and absence of glycans.

Sample	Galectin-3	Saccharide	Size Distribution (% Mass)§								Polydispersity index
			<5 nm				<100 nm		>100 nm		
			nm	%	nm	%	nm	%	nm	%	
1	CRD BF	-	2.302	99.3	-	-	48.84	Negl	324.9	0.7	0.743
2	CRD AF*	-	2.163	99.9	-	-	-	-	201.7	Negl	0.557
3	CRD AF	Lactose/2 mM	2.663	99.6	-	-	-	-	440.7	Negl	0.476
4	CRD AF	LNnT/140 µM	2.313	99.6	-	-	-	-	470.6	Negl	0.705
5	FL BF	-	3.621	33	-	-	-	-	291	67	0.785
6	FL AF*	-	3.318	99.6	-	-	-	-	309.2	Negl	0.471
7	FL AF	Lactose/8 mM	3.155	99.6	-	-	-	-	223.4	Negl	0.533
8	FL AF	LNnT/140 µM	-	-	-	-	46.73	8.7	326.5	91.3	0.627
9	FL AF	LNnT/70 µM#	3.147	66.7	4.643	33.3	-	-	-	-	0.822

Galectin-3: **CRD** Carbohydrate Recognition Domain, **FL** full length, **BF** before Filtration, **AF** after Filtration. AF* sample used for interaction studies. **Negl** Negligible. **§**Size distribution from 3 measurements of 11runs. **#**The oscillation of size throughout 9 measurements of 11 runs.

### Structural analysis of full length Galectin-3

**Figure 3:**
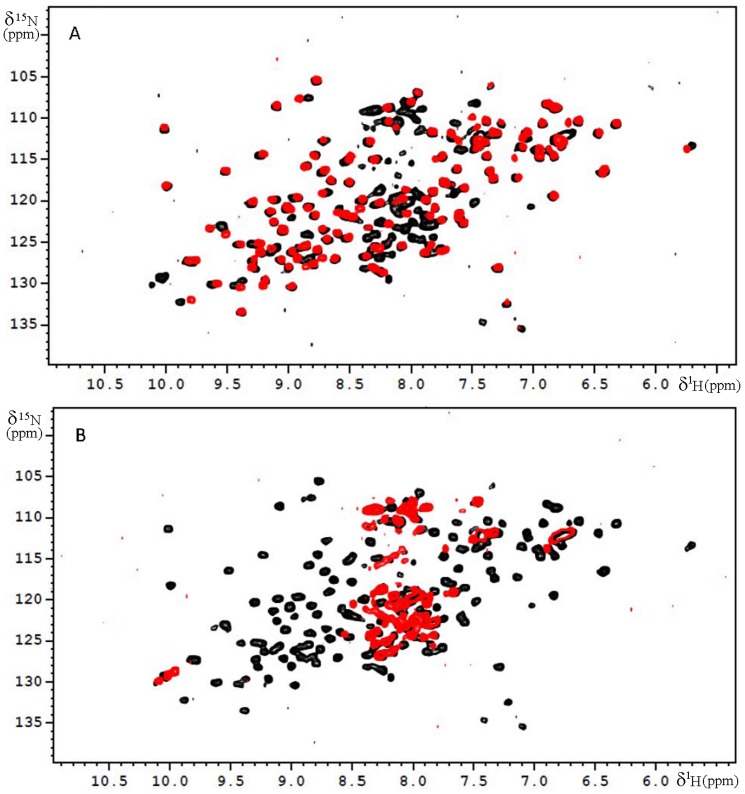
^1^H-^15^N HSQC spectra carried out on a 500 MHz NMR spectrometer at 300 K. A] ^15^N-HSQC of full length Galectin-3 (in black) at 30 µM concentration and recombinant CRD (in red) at 40 µM concentration, B] ^15^N-HSQC of full length Galectin-3 (in black) at 30 µM concentration and recombinant N-terminal domain (in red) at 30 µM concentration.

To investigate the structural features of Galectin-3 ligand-induced oligomerization, we produced ^15^N-labelled N-terminal domain, CRD domain and full length Galectin-3 to perform an NMR structural study (Fig. 3).

^1^H-^15^N HSQC spectra of the free CRD domain of Galectin-3 (Fig. 3A) is in agreement with the already published assignment of this protein [13]. The ^1^H-^15^N HSQC of the free N-terminal domain (Fig. 3B) is characteristic of an unfolded domain with resonances between 8.5 and 7.5 ppm. The ^1^H-^15^N HSQC of the full length protein is the sum of the ^1^H-^15^N HSQC spectra of the N-terminal domain and of the CRD with slight chemical shift variations in perfect agreement with the already published assignment of the full length protein [16]. In the three proteins (CRD, FL and N-terminal domain), the 6His-Tag was not assigned.

**Figure 4:**
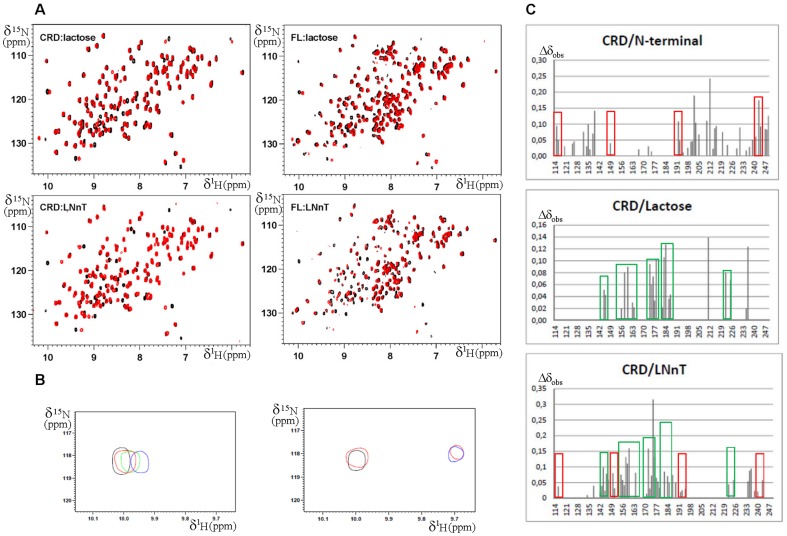
Complex formation involving Galectin-3. A] ^15^N-HSQC spectra carried out on a 600 MHz NMR spectrometer equipped with a TCI cryoprobe at 300 K. Top left, free CRD at 40 µM concentration in black and CRD at 40 µM concentration in the presence of lactose at 1.5 mM in red; top right, free full length Galectin-3 (FL) at 30 µM concentration in black and FL at 30 µM concentration in the presence of lactose at 1.5 mM in red; bottom left, CRD at 40 µM concentration in black and CRD at 40 µM concentration in the presence of LNnT at 80 µM in red; bottom right, free full length Galectin-3 (FL) at 30 µM concentration in black and FL at 30 µM concentration in the presence of LNnT at 80 µM in red. B] Zoom in of HSQC: in left box, free CRD at 40 µM concentration in black and CRD at 40 µM concentration in the presence of lactose at 0.1 mM in red, 0.8 mM in green and 1.5 mM in blue; in the right box, CRD at 40 µM concentration in black and CRD at 40 µM concentration in the presence of LNnT at 28 µM in red and 90 µM in blue. C] Chemical shift variations observed on Galectin-3 CRD NH, top, induced by the N-terminal domain within FL (Table S2); center, induced by lactose (Table S3); bottom, induced by LNnT (Table S4). Values shown were calculated using the equation Δδ_obs_ =  [(Δδ_HN_^2^ + Δδ_N_^2^/25)]^1/2^. In green boxes are highlighted residues affected by lactose. In red boxes are highlighted residues affected by the N-terminal domain within the full length protein.

**Figure 5:**
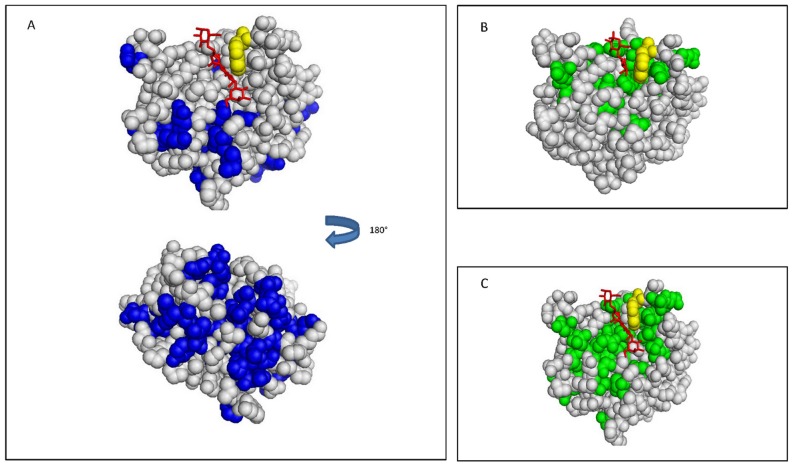
Chemical shift mapping representation on Galectin-3 CRD structure. A] Effects of the N-terminal domain on the NH groups of amino acids of the CRD. The structure of CRD in the presence of LNnT (PDB 4LBN) is shown. In yellow is Trp181, in red is LNnT and in blue are the amino acids of the CRD with chemical shift variations in the presence of the N-terminal domain within the full length Galectin-3. B] Effects of lactose on the NH groups of amino acids of the CRD. The structure of CRD in the presence of lactose (PDB 3ZSJ) is shown. In yellow is Trp181, in red is the lactose and in green are amino acids of CRD with chemical shift variations induced by the presence of lactose. C] Effects of LNnT on the NH groups of amino acids of the CRD. The structure of CRD in the presence of LNnT (PDB 4LBN) is shown. In yellow is Trp181, in red is LNnT and in green are amino acids of the CRD with chemical shift variations induced by the presence of LNnT.

To access the interactions between the CRD and N-terminal domains we compared ^1^H-^15^N HSQC spectra of the free CRD with that of CRD within the FL (Fig. 4C and Fig. 5A). Chemical shift variation analysis indicates that the markedly shifted resonances of CRD belong to some residues close to the sugar binding site and residues located at the backside of the lectin (residues Ile132, Leu135, Val138, Lys139, Phe192, Glu193, Phe198, Ile200, Gln201, Val202, Leu203, Glu205, Lys210, Ala212, Asp215, Ala216, Asp241, Thr243 and Ser244) (Fig. 5A). This result is in perfect agreement with the peptide analysis already reported by NMR spectroscopy indicating that the N-terminal domain of Galectin-3 interacts with residues of CRD located at the back of the molecule [17]. Moreover, residues located at the N- and C-terminal extremities of the CRD (residues Ile115 and Val116, and Tyr247, Thr248 and Met249) were also perturbed by the presence of the N-terminal domain as previously predicted by modeling of Galectin-3 involving the β-strands S1 and S12 [18]. The binding of the N-terminal domain of Galectin-3 on the N- and C-terminal segments of the CRD may explain the monomeric status of this galectin in solution.

On the other hand, comparison of ^1^H-^15^N HSQC of the N-terminal domain alone and within the full length Galectin-3 was more difficult to interpret (Fig. 3B). Even if the assignment of the full length Galectin-3 has been recently reported [16], it was not possible to give a chemical shift mapping of the CRD domain interacting zone on the N-terminal unstructured domain in full length Galectin-3. Due to the various conformations of the flexible N-terminal domain, the presence of repeat segments and the poor resolution of the NMR spectra of the N-terminal domain, unambiguous sequence specific assignments within the complex were difficult to obtain. However, we could observe that numerous resonances of the N-terminal domain underwent perturbations when comparing the spectra of the N-terminal domain alone and within the full length protein, confirming that the N-terminal domain interacts with the CRD (Fig. 3A).

### Structural analysis of Galectin-3 oligosaccharide complexes

#### 1. Complex formation of CRD and full length Galectin-3 with lactose

**Figure 6:**
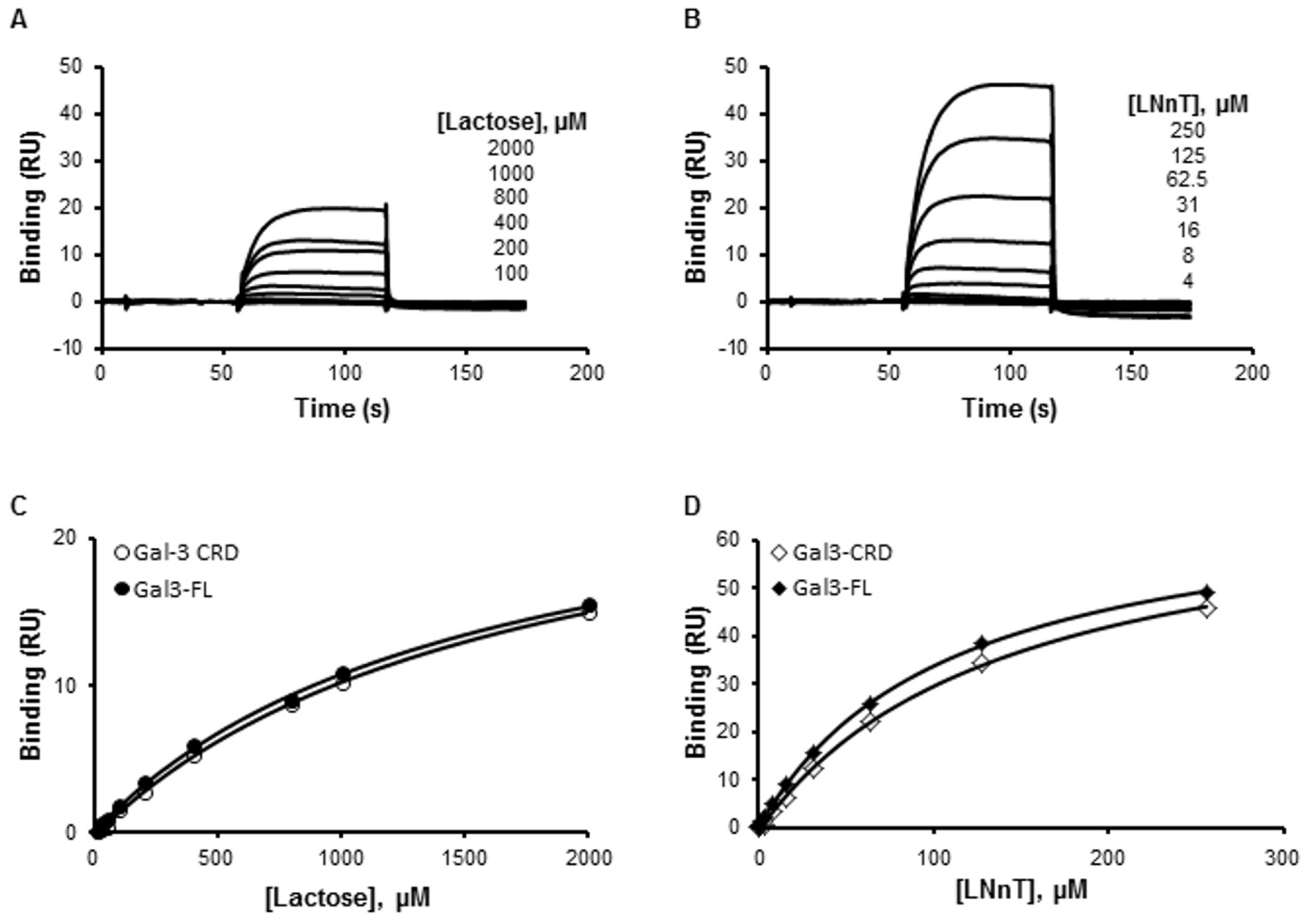
SPR measurement of lactose and LNnT binding to CRD and FL. Increasing concentrations of lactose (12.5–2000 µM, **A, C**) and LNnT (0.5–250 µM, **B, D**) were injected over immobilized CRD and FL. Typical sensorgrams obtained with CRD (**A**, **B**) are displayed. The measured plateau values were used to plot saturation binding curves (**C**, **D**). These curves were used to calculate the K_D_ value for lactose and LNnT bindings to CRD and FL. One curve is representative of at least two independent experiments.

Figures 4 and 5B show the NMR titrations conducted on the CRD with lactose. The residues for which we observed chemical shift variations correspond to ones shown in the x-ray structure to be involved in the formation of the CRD/lactose complex (PDB 3ZSJ). No significant difference was observed between the affected residues of the CRD alone and full length protein. This indicated that lactose does not affect N-terminal domain/CRD interactions (Fig. 4A and 4C). The K_D_ obtained by SPR measurements with lactose (Fig. 6) were in agreement with our NMR data as similar affinities for CRD (1.25±0.18 mM) and for FL (1.12±0.2 mM) were observed. These results clearly established that the N-terminal domain did not mediate nor enhance lactose/CRD interactions.

#### 2. Complex formation of CRD and full length Galectin-3 with LNnT

Galectin-3 is known to interact with polyLacNAc oligosaccharides [14] and among them the structure of CRD/LNnT complex was solved by x-ray (PDB 4LBN). SPR experiments show that both the CRD and the FL proteins have a higher affinity for LNnT than for lactose (Fig. 6). Moreover, the affinities of CRD and FL were similar for LNnT indicating that the N-terminal domain does not mediate the lectin/LNnT interaction (0.14±0.017 mM for CRD and 0.12±0.016 mM for FL). The slow exchange of NMR chemical shift variations observed in the presence of LNnT and the fast exchange observed in the presence of lactose were also in agreement with a higher affinity for LNnT than for lactose (Fig. 4B). X-ray structural data on CRD/ligand interactions (PDB 3ZSJ and 4LBN) revealed that the binding of the core β-galactoside is highly conserved in all analyzed complexes. In particular, the interactions formed by Trp181 with O4 and O6 of the galactose moiety are always observed. ^1^H-^15^N HSQC spectra indicate that the ligand affects the CRD and FL spectra in a similar way (Fig. 4C and Fig. 5C). In the crystal structure of CRD/LNnT complex [19], there is a well-defined electron density for the three carbohydrate residues from the reducing end of LNnT (GlcNacβ1-3Galβ1-4Glc). These residues form identical hydrogen bonding with the protein as those in the lactose-bound structures. The GlcNac residue that is β1-3 linked to the lactose in the tetrasaccharide extends the Galectin-3 binding site by the formation of direct hydrogen bonds with the protein side chains (Arg144 and Asp148). Consistently, these two residues show additional chemical shift variations in the spectrum of CRD in the presence of LNnT (Fig. 4C). In addition, water- mediated interactions are observed between GlcNac carbonyl oxygen and the side chains of Arg144 and Asn160, and van der Waals contacts are found between GlcNac C6/O6 and Asp148 and His158. Additional chemical shifts are observed for these residues in the NMR spectra in the presence of LNnT (Fig. 4C). The terminal β1-4 galactose residue is relatively poorly defined in the electron density and it forms weak interactions with Gly238 and Arg144 in agreement with the additional chemical shifts observed in the NMR spectra in the presence of LNnT (Fig. 4C). It is clear that these favorable interactions are consistent with a greater affinity for LNnT than for lactose, as calculated from SPR experiments (0.14±0.017 mM for LNnT and 1.25±0.18 mM for lactose with CRD) (Fig. 6). As mentioned in the literature, the conformation of LNnT and its contact at the surface of the protein limits the types of extensions suitable for Galectin-3 ligands. Any extension would, however, lie along the binding groove [14]. When comparing the effect of LNnT binding on the quality of the NMR spectra of CRD and full length Galectin-3, one can observe decreased peak intensity in the NMR spectra of the full length protein after LNnT addition (Fig. 4A). Such decrease for the bound protein is correlated to a glycan induced oligomerization observed in DLS experiments for the FL protein but not for the CRD alone, and results in a lower concentration of the soluble form. We thus concluded that LNnT induces a full length Galectin-3 N-type self-association.

### Structural implications of N-type self-oligomerization of Galectin-3

On the basis of our NMR titrations, we analyzed the effects induced by the N-terminal domain, lactose and LNnT on the CRD chemical shifts (Fig. 4C). One can observe that the chemical shift variations on the CRD in the presence of LNnT are the sum of those induced by lactose and some due to the N-terminal domain. Thus, the N-terminal interface and the LNnT interface overlap on the CRD, indicating that one interaction might alter the other (Fig. 5). These data bring us to conclude that LNnT removes the N-terminal domain from the CRD interface by competition, triggering the release of this N-terminal domain and resulting in the oligomerization of the full length Galectin-3 *via* a N-type glycan-dependent self-association.

At the cell surface when Galectin-3 is overexpressed, two independent Galectin-3 self-association processes are the driving force of lattice formation. The first is a glycan-dependent N-type association where Galectin-3 forms heterogeneous oligomers through N-terminal domain interactions. The second is glycan-independent and also observed for the CRD alone, thus defined as C-type association. In the full length galectin-3, the C-type association is probably enhanced by the N-type association. In this work, we show how in the presence of LNnT, N-type oligomerization increases the exposure of the N- and C-terminal extremities of the CRD domain which are favorable to the C-type interactions. These two driving forces assisted by the specific positioning of glycans at the cell surface may induce a proper lattice formation. Such lattice is necessary to strengthen cell-cell interactions under dragging forces imposed by the fluid flow acting on cells or bacteria. The protein-protein interactions encountered at the N-terminal domain/CRD interface of Galectin-3 may be considered as new potential targets for drug design in cancer.

## Supporting Information

Table S1 (DOCX)

Table S2 (DOCX)

Table S3 (DOCX)

Table S4 (DOCX)
