# Supplementary material for: Glycan Dependence of Galectin-3 Self-Association Properties
Source: PLoS One. 2014 Nov 4;9(11):e111836. doi: 10.1371/journal.pone.0111836 (PMC4219786; doi:10.1371/journal.pone.0111836)
Supplement: Table S1 — (DOCX) [file pone.0111836.s001.docx]

**Table S1 : Sequences of 5’ and 3’ primers used for His-tag PCR amplification.** The restriction sites *Nde*I and *BamH*I are in bold type and the sequence encoding the 6His-tag is in italics

|  | 5’ primers | 3’ primers |
| --- | --- | --- |
| 6His-N-ter (13-113) | 5'-GGAATTC**CATATG***CATCATCATCATCATCAT*G  GGTCTGGAAACCCAAACCCTCAAGGATG-3' | 5'- CC**GGATCC**TTATGGGGCGCCATA-3' |
| CRD (114-250)-6His | 5′-CCGC**CATATG**CTGATTGTGCCTTATAACCTG-3′ | 5′-CG**GGATCC**TTA*ATGATGATGATGATGATG*TATC-3′ |
| FL (1-250)-6His | 5′-CCGC**CATATG**GCAGACAATTTTTCGCTCCATG-3′ | 5′-CG**GGATCC**TTA*ATGATGATGATGATGATG*TATC-3′ |
