# Supplementary material for: Glycan Dependence of Galectin-3 Self-Association Properties
Source: PLoS One. 2014 Nov 4;9(11):e111836. doi: 10.1371/journal.pone.0111836 (PMC4219786; doi:10.1371/journal.pone.0111836)
Supplement: Table S2 — (DOCX) [file pone.0111836.s002.docx]

**Table S2**: Chemical shifts of H_N_ and N_H_ for CRD and FL Galectin-3. Δδobs is the observed chemical shift variations calculated using the equation Δδ_obs_ = [(Δδ_HN_ ^2^ +Δδ_N_^2^/25)]^1/2^

| aa | CRD H_N_ | CRD N_H_ | FL H_N_ | FL N_H_ | Δδobs |
| --- | --- | --- | --- | --- | --- |
| LEU114 | 0 | 0 | 0 | 0 | 0.00 |
| ILE115 | 8.18 | 122.92 | 8.24 | 122.56 | 0.09 |
| VAL116 | 7.84 | 121.89 | 7.89 | 121.88 | 0.05 |
| PRO117 |  |  |  |  | 0.00 |
| TYR118 |  |  |  |  | 0.00 |
| ASN119 | 7.74 | 126 | 7.74 | 126 | 0.00 |
| LEU120 | 9.3 | 128.18 | 9.27 | 128.18 | 0.03 |
| PRO121 |  |  |  |  | 0.00 |
| LEU122 | 7.75 | 122.35 | 7.75 | 122.35 | 0.00 |
| PRO123 |  |  |  |  | 0.00 |
| GLY124 |  |  |  |  | 0.00 |
| GLY125 | 8.207 | 108.86 | 8.17 | 108.81 | 0.04 |
| VAL126 | 7.08 | 113.67 | 7.09 | 113.45 | 0.05 |
| VAL127 | 6.43 | 116.73 | 6.43 | 116.73 | 0.00 |
| PRO128 |  |  |  |  | 0.00 |
| ARG129 | 8.7 | 112.77 | 8.7 | 112.77 | 0.00 |
| MET130 | 7.83 | 118.94 | 7.83 | 118.94 | 0.00 |
| LEU131 | 8.99 | 128.04 | 8.99 | 128.04 | 0.00 |
| ILE132 | 9.8 | 132.1 | 9.87 | 132.24 | 0.08 |
| THR133 |  |  |  |  | 0.00 |
| ILE134 | 9.58 | 130.11 | 9.61 | 130.11 | 0.03 |
| LEU135 | 8.72 | 127 | 8.81 | 126.8 | 0.10 |
| GLY136 | 6.88 | 108.35 | 6.89 | 108.26 | 0.02 |
| THR137 | 8.86 | 116 | 8.86 | 116 | 0.00 |
| VAL138 | 8.3 | 128.18 | 8.37 | 128.16 | 0.07 |
| LYS139 | 8.22 | 128.79 | 8.18 | 129.47 | 0.14 |
| PRO140 |  |  |  |  | 0.00 |
| ASN141 |  |  |  |  | 0.00 |
| ALA142 | 6.83 | 119.46 | 6.83 | 119.46 | 0.00 |
| ASN143 | 9.5 | 116.45 | 9.5 | 116.45 | 0.00 |
| ARG144 | 8.5 | 117.8 | 8.5 | 117.8 | 0.00 |
| ILE145 | 8.06 | 119.83 | 8.06 | 119.83 | 0.00 |
| ALA146 | 8.62 | 127.14 | 8.62 | 127.14 | 0.00 |
| LEU147 | 8.46 | 121.95 | 8.46 | 121.95 | 0.00 |
| ASP148 |  |  |  |  | 0.00 |
| PHE149 | 9.23 | 125.22 | 9.27 | 125.22 | 0.04 |
| GLN150 | 9.05 | 127.18 | 9.05 | 127.18 | 0.00 |
| ARG151 | 8.06 | 125.48 | 8.06 | 125.48 | 0.00 |
| GLY152 |  |  |  |  | 0.00 |
| ASN153 |  |  |  |  | 0.00 |
| ASP154 | 8.4 | 119.88 | 8.4 | 119.88 | 0.00 |
| VAL155 | 9.13 | 121.33 | 9.13 | 121.33 | 0.00 |
| ALA156 | 8.8 | 127.7 | 8.8 | 127.7 | 0.00 |
| PHE157 | 7.56 | 118.54 | 7.56 | 118.54 | 0.00 |
| HIS158 | 9.38 | 133.51 | 9.38 | 133.51 | 0.00 |
| PHE159 | 8.36 | 126.79 | 8.36 | 126.79 | 0.00 |
| ASN160 |  |  |  |  | 0.00 |
| PRO161 |  |  |  |  | 0.00 |
| ARG162 | 9.23 | 126.35 | 9.23 | 126.35 | 0.00 |
| PHE163 | 8.04 | 118.7 | 8.04 | 118.7 | 0.00 |
| ASN164 | 8.65 | 117.58 | 8.65 | 117.58 | 0.00 |
| GLU165 | 8.88 | 125.65 | 8.88 | 125.65 | 0.00 |
| ASN166 |  |  |  |  | 0.00 |
| ASN167 | 7.99 | 108.15 | 8.01 | 108.13 | 0.02 |
| ARG168 | 7.32 | 117.35 | 7.32 | 117.35 | 0.00 |
| ARG169 | 7.87 | 120 | 7.87 | 120 | 0.00 |
| VAL170 | 8.53 | 121.67 | 8.53 | 121.67 | 0.00 |
| ILE171 | 8.68 | 124.7 | 8.68 | 124.7 | 0.00 |
| VAL172 | 7.28 | 128.14 | 7.28 | 128.14 | 0.00 |
| CYS173 | 8.72 | 123.44 | 8.69 | 123.46 | 0.03 |
| ASN174 | 8.91 | 119.62 | 8.91 | 119.62 | 0.00 |
| THR175 | 10 | 118.31 | 10 | 118.24 | 0.01 |
| LYS176 | 8.97 | 130.4 | 8.97 | 130.4 | 0.00 |
| LEU177 | 7.88 | 126.33 | 7.88 | 126.33 | 0.00 |
| ASP178 | 8.96 | 126.18 | 8.96 | 126.18 | 0.00 |
| ASN179 | 9.09 | 108.58 | 9.09 | 108.58 | 0.00 |
| ASN180 | 7.72 | 117.72 | 7.72 | 117.72 | 0.00 |
| TRP181 | 8.91 | 127.16 | 8.91 | 127.16 | 0.00 |
| GLY 182 | 8.17 | 110.53 | 8.17 | 110.53 | 0.00 |
| ARG183 | 8.59 | 123.91 | 8.59 | 123.91 | 0.00 |
| GLU184 | 8.76 | 125.99 | 8.76 | 125.99 | 0.00 |
| GLU185 | 9.12 | 122.56 | 9.12 | 122.56 | 0.00 |
| ARG186 | 8.87 | 125.5 | 8.87 | 125.5 | 0.00 |
| GLN187 | 8.78 | 121.77 | 8.78 | 121.77 | 0.00 |
| SER188 |  |  |  |  | 0.00 |
| VAL189 | 7.75 | 125.99 | 7.75 | 125.99 | 0.00 |
| PHE190 | 8.3 | 125.59 | 8.3 | 125.59 | 0.00 |
| PRO191 |  |  |  |  | 0.00 |
| PHE192 | 5.74 | 113.87 | 5.69 | 113.39 | 0.11 |
| GLU193 | 9.14 | 120 | 9.1 | 119.87 | 0.05 |
| SER194 | 9 | 120.74 | 9 | 120.74 | 0.00 |
| GLY195 | 7.05 | 111.86 | 7.06 | 111.85 | 0.01 |
| LYS196 | 7.72 | 117.89 | 7.72 | 117.89 | 0.00 |
| PRO197 |  |  |  |  | 0.00 |
| PHE198 | 8.7 | 116.42 | 8.72 | 116.5 | 0.03 |
| LYS199 |  |  |  |  | 0.00 |
| ILE200 | 9.75 | 127.26 | 9.79 | 127.36 | 0.04 |
| GLN201 | 9.23 | 125.1 | 9.27 | 125.22 | 0.05 |
| VAL202 | 9.51 | 124.11 | 9.53 | 123.17 | 0.19 |
| LEU203 | 9.39 | 130.62 | 9.43 | 130.14 | 0.10 |
| VAL204 | 8.91 | 127.16 | 8.91 | 127.16 | 0.00 |
| GLU205 | 8.29 | 128.27 | 8.25 | 128.54 | 0.07 |
| PRO206 |  |  |  |  | 0.00 |
| ASP207 | 7.95 | 107 | 7.95 | 107 | 0.00 |
| HIS208 | 6.42 | 116.34 | 6.42 | 116.34 | 0.00 |
| PHE209 | 8.505 | 115.118 | 8.505 | 115.118 | 0.00 |
| LYS210 | 9.64 | 123.37 | 9.54 | 123.14 | 0.11 |
| VAL211 | 8.85 | 124 | 8.85 | 124 | 0.00 |
| ALA212 | 9.2 | 130.34 | 9.37 | 129.47 | 0.24 |
| VAL213 |  |  |  |  | 0.00 |
| ASN214 | 9.81 | 127.42 | 9.79 | 127.37 | 0.02 |
| ASP215 | 8.91 | 107.77 | 8.83 | 107.61 | 0.09 |
| ALA216 | 7.6 | 121.7 | 7.58 | 122.16 | 0.09 |
| HIS217 |  |  |  |  | 0.00 |
| LEU218 |  |  |  |  | 0.00 |
| LEU219 | 7.4 | 110.4 | 7.4 | 110.4 | 0.00 |
| GLN220 | 8.72 | 119.19 | 8.67 | 118.91 | 0.08 |
| TYR221 |  |  |  |  | 0.00 |
| ASN222 | 9.19 | 129.67 | 9.19 | 129.67 | 0.00 |
| HIS223 | 7.11 | 117.21 | 7.14 | 117.29 | 0.03 |
| ARG224 |  |  |  |  | 0.00 |
| VAL225 | 8.26 | 120.34 | 8.26 | 120.34 | 0.00 |
| LYS226 |  |  |  |  | 0.00 |
| LYS227 | 7.03 | 120.75 | 7.03 | 120.75 | 0.00 |
| LEU228 | 7.57 | 122.77 | 7.57 | 122.77 | 0.00 |
| ASN229 | 8.33 | 112.9 | 8.35 | 112.96 | 0.02 |
| GLU230 | 7.35 | 116.45 | 7.35 | 116.45 | 0.00 |
| ILE231 | 7.62 | 121.78 | 7.57 | 122.15 | 0.09 |
| SER232 | 7.62 | 116.18 | 7.62 | 116.18 | 0.00 |
| LYS233 | 7.8 | 120.84 | 7.8 | 120.84 | 0.00 |
| LEU234 | 8.5 | 124.4 | 8.5 | 124.4 | 0.00 |
| GLY235 | 9.21 | 114.51 | 9.22 | 114.58 | 0.02 |
| ILE236 | 9.27 | 127.11 | 9.27 | 127.11 | 0.00 |
| SER237 | 9.28 | 120.2 | 9.3 | 120.3 | 0.03 |
| GLY238 | 8.77 | 105.5 | 8.77 | 105.5 | 0.00 |
| ASP239 | 7.08 | 112.55 | 7.03 | 112.52 | 0.05 |
| ILE240 | 7.82 | 111.73 | 7.82 | 111.73 | 0.00 |
| ASP241 | 8.83 | 121 | 8.82 | 120.7 | 0.06 |
| LEU242 |  |  |  |  | 0.00 |
| THR243 | 8.98 | 121.2 | 8.97 | 122.07 | 0.17 |
| SER244 | 7.68 | 111.93 | 7.67 | 112.39 | 0.09 |
| ALA245 | 8.3 | 125.59 | 8.3 | 125.59 | 0.00 |
| SER246 | 8.49 | 114.71 | 8.49 | 114.71 | 0.00 |
| TYR247 | 8.3 | 115.17 | 8.25 | 114.83 | 0.08 |
| THR248 | 8.78 | 114.7 | 8.77 | 114.29 | 0.08 |
| MET249 | 8.59 | 121.54 | 8.57 | 120.92 | 0.13 |
| ILE250 |  |  |  |  | 0.00 |
